# Residual HIV-1 DNA Flap-independent nuclear import of cPPT/CTS double mutant viruses does not support spreading infection

**DOI:** 10.1186/1742-4690-8-92

**Published:** 2011-11-10

**Authors:** Candela Iglesias, Mathieu Ringeard, Francesca Di Nunzio, Juliette Fernandez, Raphael Gaudin, Philippe Souque, Pierre Charneau, Nathalie Arhel

**Affiliations:** 1 Molecular Virology and Vaccinology Unit, Department of Virology, Institut Pasteur, Paris, France; 2 Centro de Investigacion en Enfermedades Infecciosas, Mexico Tlalpan, Mexico; 3 Molecular Virology Laboratory, Institut de Génétique Humaine, Montpellier, France; 4 Immunity and Cancer Unit, Institut Curie, Paris, France

## Abstract

**Background:**

The human immunodeficiency virus type 1 (HIV-1) central DNA Flap is generated during reverse transcription as a result of (+) strand initiation at the central polypurine tract (cPPT) and termination after a *ca*. 100 bp strand displacement at the central termination sequence (CTS). The central DNA Flap is a determinant of HIV-1 nuclear import, however, neither cPPT nor CTS mutations entirely abolish nuclear import and infection. Therefore, to determine whether or not the DNA Flap is essential for HIV-1 nuclear import, we generated double mutant (DM) viruses, combining cPPT and CTS mutations to abolish DNA Flap formation.

**Results:**

The combination of cPPT and CTS mutations reduced the proportion of viruses forming the central DNA Flap at the end of reverse transcription and further decreased virus infectivity in one-cycle titration assays. The most affected DM viruses were unable to establish a spreading infection in the highly permissive MT4 cell line, nor in human primary peripheral blood mononuclear cells (PBMCs), indicating that the DNA Flap is required for virus replication. Surprisingly, we found that DM viruses still maintained residual nuclear import levels, amounting to 5-15% of wild-type virus, as assessed by viral DNA circle quantification. Alu-PCR quantification of integrated viral genome also indicated 5-10% residual integration levels compared to wild-type virus.

**Conclusion:**

This work establishes that the central DNA Flap is required for HIV-1 spreading infection but points to a residual DNA Flap independent nuclear import, whose functional significance remains unclear since it is not sufficient to support viral replication.

## Background

During infection, the reverse transcriptase (RT) of retroviruses converts the (+) strand RNA genome into double-stranded DNA prior to nuclear import. Similar to all DNA polymerases, the retroviral RT requires the 3' OH of a primer to initiate polymerisation. The primer used for (-) strand synthesis is a cellular tRNA already present in the retroviral particles prior to infection and anneals to the 5' region of the genome at the primer binding site (PBS). The primer used for (+) strand synthesis is a polypurine tract (PPT) present in the 3' region of the RNA genome that resists RNase H degradation concomitant with (-) strand synthesis. The genome of lentiviruses contains two such cis-acting purine-rich sequences: the 3'PPT common to all retroviruses and an additional central PPT present in the coding sequence of the integrase. The resulting initiation of (+) strand synthesis at the cPPT as well as at the 3'PPT leads to a (+) strand discontinuity at the centre of lentiviral genomes [1-5].

Similar to central (+) strand initiation, the termination of reverse transcription is a further feature that distinguishes lentiviruses from other orthoretroviruses. For most retroviruses, termination occurs when the RT reaches the 5' end of the template. In the case of lentiviruses however, such as HIV-1 [6], EIAV [4], and FIV [5], reverse transcription terminates *ca*. 100 nt downstream of the cPPT at stretches of A and T nucleotides (the central termination sequence, CTS) whose conformation disfavours binding to RT enzyme and thus halts reverse transcription. As a result of the cPPT and CTS cis-acting sequences, the final product of lentiviral reverse transcription contains a *ca*. 100 nt overlap, or "DNA Flap", at the centre of the genome.

Mutations introduced either in the cPPT [7-9] or in the CTS [6] of HIV-1 lead to a loss of HIV-1 infectivity that is proportional to the number and positional impact of introduced mutations. The conversion of cPPT purines into pyrimidines compromises central initiation of (+) strand synthesis and thus central DNA Flap formation [7]. Similarly, the mutation of dA*_n_*-dT*_n _*tracts in the CTS leads to aberrant (+) strand termination events spread over a distance of over 500 nt downstream of the cPPT [6]. Therefore, both the cPPT and CTS are required to form a functional central DNA Flap, and HIV-1 infection is impaired if either are mutated.

The defect in viral replication observed for cPPT mutant viruses [7,10,11] is not accounted for by defects in viral DNA synthesis, integration, or virus production [9] but by a defect in nuclear import [9,10,12]. The importance of the central DNA Flap for nuclear import was harnessed by the gene therapy field when it was shown that re-insertion of the cPPT and CTS sequences in lentiviral vectors significantly enhanced gene transfer efficiencies by increasing nuclear import [9,13]. It is now systematically included in lentiviral vectors.

Interestingly, central (+) strand initiation has been shown to protect the HIV-1 genome from editing by cytidine deaminases of the APOBEC3 family downstream of the cPPT sequence [14]. Protection was high closest to the cPPT and decreased progressively further downstream. APOBEC3 enzymes edit exclusively single-stranded DNA, which in the case of HIV corresponds to the newly-synthesised (-) strand DNA. Editing is possible only until synthesis of the complementary (+) strand. Therefore, sequences immediately downstream of (+) strand initiation PPT sequences are more protected from editing than those upstream or further downstream [14,15]. It has been suggested that APOBEC3G/F editing wholly accounts for the infectivity defect of cPPT mutant HIV [16]. However, the benefit of the central DNA Flap is not contested in the context of HIV-1-derived vectors, which are standardly produced in APOBEC3G-negative 293T cells [5,9,10,12,13,17-34]. Furthermore, in the case of CTS mutants, a (+) strand segment is correctly initiated at the cPPT [6] and should protect downstream sequences from deamination as in wild-type virus. Therefore, the observed strong replication defect of CTS mutants in Vif non-permissive cells [16] cannot be attributed to APOBEC3G restriction.

Nevertheless, the importance of the central DNA Flap for HIV-1 nuclear import, infectivity and replication *in vivo *has been a source of much debate over the past 10 years. Some papers have proposed that the central DNA Flap contributes minimally, if at all, to HIV-1 replication, although some degree of impairment for DNA Flap mutants is observed in human PBMCs and macrophages [16,35-38]. However, other studies claim that the central DNA Flap plays an important role in HIV nuclear import, infectivity, transduction efficiency, and viral replication [6,7,9-13,17-34,39]. Therefore, the debate seems really to reside in whether the central DNA Flap is essential for infection and replication, or whether it merely contributes towards rendering these more efficient.

Mutations in the cPPT do not lead to totally non-infectious viruses [6,7] and some residual HIV-1 nuclear import is observed [10], suggesting either that viruses may enter the nucleus in a DNA-Flap-independent manner or that the mutations introduced in the cPPT are not sufficient to abolish DNA Flap formation. Unfortunately, the extent of mutations that can be introduced in the cPPT is limited by the need to maintain a functional integrase. The 225T mutant [17], which contains 6 synonymous mutations, maintains residual infectivity probably because the cPPT is only partially inactivated. To more profoundly disrupt DNA Flap formation, the cPPT-D mutant was generated to contain an additional K→R amino acid change and compared with a control virus cPPT-AG containing the same K→R mutation, but maintaining the polypurine nature of the cPPT [9]. Despite non-detectable (+) strand discontinuity, the cPPT-D mutant still retained residual infectivity and nuclear import (5-10% of wild-type HIV-1) [9]. Therefore, it remains an open question whether observed residual infection is due to only partial disruption of the DNA Flap, or whether a DNA-Flap-independent nuclear import exists in *ca*. 10% of infectious events. Since we cannot introduce any further mutations in the cPPT, we set out to combine cPPT mutations with the previously described CTS mutant that leads to aberrant (+) strand termination [6], on the premise that residual central strand initiation will then not lead to functional DNA Flap formation. We thus report the generation of fully disruptive mutants for DNA Flap formation that preserve integrase function, and document their effect on nuclear import, infectivity and replication.

## Results

### Production of wild-type and mutant viruses

**Figure 1:**
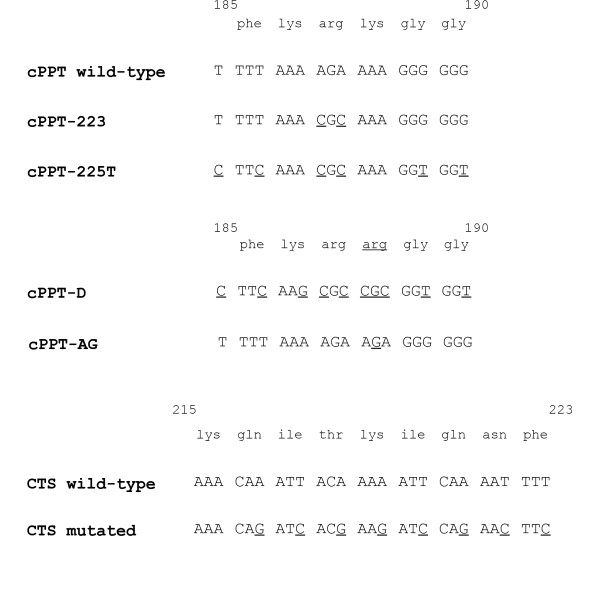
**Mutations introduced in the cPPT and/or CTS regions of HIV-1_LAI_**. The 223 mutation introduces two pyrimidines in the A-boxes, and the 225T mutant is derived from the 225 mutant, which contains two additional pyrimidines in the G-box. The T mutation refers to the introduction of two cytosines in the T-box. The cPPT-D mutation [9] corresponds to the 225T mutant with 4 additional mutations in A-boxes, totalling to 10 mutations in the 19-mer cPPT. The CTS mutant has a total of 8 mutations in the *ter1 *and *ter2 *domains [6]. Numbers indicate amino acid positions in HIV-1 IN.

In HIV-1, the cPPT sequence is an exact copy of the 3'PPT and is composed sequentially of a T-box, two A-boxes and a G-box (Figure 1). We previously found that the two A-boxes have the most profound impact on HIV-1 infectivity when mutated, followed in importance by the G- and lastly the T-box (P. Charneau, unpublished data). The 223 mutant, which introduces two pyrimidines in the A-boxes, is one of the least affected mutants [7]. The 225T mutant combines mutations in all four boxes and is the most affected amongst the synonymous cPPT mutants [17]. The addition of a single non-synonymous mutation (cPPT-D mutant) has the most profound effect on viral replication [9]. Although this mutant introduces an amino acid change at position 188 of the integrase coding region (K188R), the control virus (cPPT-AG) with the same amino acid change behaved like wild-type virus in terms of virus production and viral DNA synthesis [9]. Position 188 of integrase (IN) is important for salt bridge formation between the catalytic core and the N-terminal domain, however, the presence of an arginine rather than a lysine at this position is naturally found in HIV-2 IN and does not perturb salt bridge formation [40]. The previously described CTS mutant, which was designed to disrupt the dA*_n_*-dT*_n _*tracts of the CTS while respecting the corresponding integrase amino acid sequence, results in DNA Flaps of abnormal length and severely impairs infectivity [6].

We generated double mutants (DM) by combining in HIV-1_LAI _and HIV-1_LAI _Δenv-vsvG viruses, the 223, 225T or cPPT-D cPPT mutants with the previously described CTS mutation [6]. There were no appreciable differences in virus p24 concentration between the wild-type virus and any of the mutant viruses **(**Additional File 1 Figure S1), confirming that DNA Flap mutations do not affect virus production [9].

### Impact of DNA Flap mutations in single-cycle infectivity

**Figure 2:**
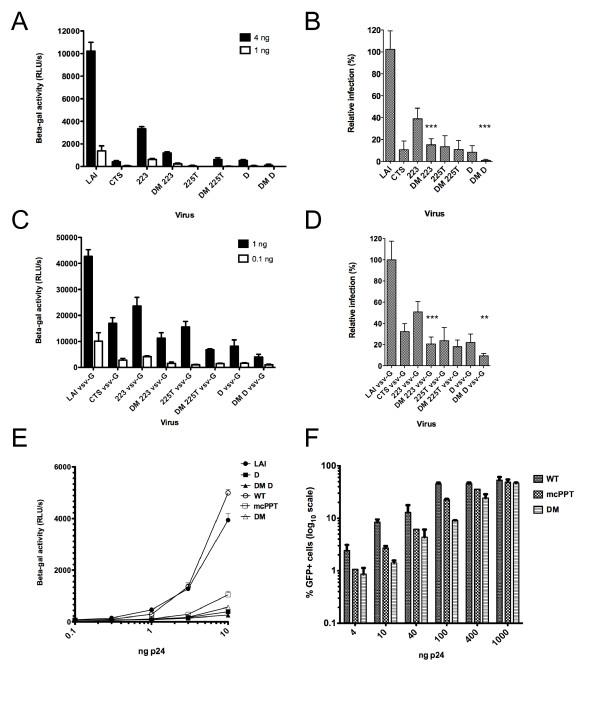
**Single-cycle titration assay in P4-CCR5 cells indicate an increased infectivity defect of DNA Flap double mutant viruses compared with cPPT or CTS single mutants**. **(A) **P4-CCR5 cells were infected with 4 ng or 1 ng p24 of HIV-1 wild-type envelope and infectivity was measured 48 h p.i by tat transactivation of the beta-gal promoter (mean of triplicates +/- SD representative of two independent experiments). **(B) **Relative infection of the DNA Flap mutant viruses compared with wild-type LAI virus (mean of three independent experiments carried out in triplicate over a range of viral concentrations +/- SD). Statistical relevance was calculated using paired t test to compare cPPT single mutant viruses with their corresponding DM virus, *** p < 0.001. **(C) **P4-CCR5 cells were infected with 1 ng or 0.1 ng p24 of HIV-1 VSV-G and infectivity was measured 48 h p.i by Tat transactivation of the beta-gal promoter (mean of triplicates +/- SD representative of three independent experiments). **(D) **Relative infection of the DNA Flap mutant viruses compared with wild-type LAI virus (mean of three independent experiments carried out in triplicate +/- SD). Statistical relevance was calculated using paired t test to compare cPPT single mutant viruses with their corresponding DM virus, *** p < 0.001. **(E) **P4-CCR5 cells were infected with wild-type env NL4-3 viruses from the Hu et al., 2010 study (WT, mcPPT, DM) in parallel with our wild-type and mutant LAI viruses (LAI, D, DM D). Results show the mean beta-galactosidase activity of duplicates +/- SD and are representative of three independent experiments. **(F) **GHOST3 cells were infected with increasing doses of viruses from the Hu et al. study [16]. Infectivity was assessed at 48 h p.i using flow cytometry. Results show the mean percentage of GFP positive cells of three independent experiments.

We first tested the single and double mutant viruses for infectivity in single cycle infections in the P4-CCR5 reporter cell line, which are HeLa CD4+ CXCR4+ CCR5+ carrying the Lac Z gene under the control of the HIV-1 LTR promoter [6]. cPPT and CTS HIV-1 mutants exhibited an impairment of infection that was significant and increasingly important with increasing number of mutations (Figure 2A). Reduction was consistent and was of 2-, 14-, 16- and 20-fold for 223, CTS, cPPT-D and 225T, respectively, compared to wild-type virus (Figure 2B). The combination of cPPT and CTS mutations increased this defect significantly in the case of the DM-223 and DM-D mutants. The DM-D mutant was the most affected of all DNA Flap mutants and only retained a very residual infectivity of 0.8 +/- 0.3% of wild-type virus (Figure 2B). The pseudotyping of HIV-1 with the vesicular stomatitis virus glycoprotein (VSV-G), which confers broad tropism and increases infectivity by 10-fold, attenuated the infectivity defects observed with wild-type envelope HIV-1_LAI _viruses (Figure 2C). The average reduction in infectivity was between 2- and 5-fold for all single mutants. However, the combining of cPPT and CTS mutations significantly further decreased the infectivity of pseudotyped viruses. The most affected mutant, DM-D VSV-G, retained an infectivity of 9.4 +/- 0.9% of wild-type virus (Figure 2D).

Taken together, these results indicate that all cPPT single and double mutants are severely impaired in infectivity, but that the most disrupted mutant still maintains residual infection (*ca*. 1% for non-pseudotyped viruses and *ca*. 10% for VSV-G pseudotyped viruses) in a one-round titration assay.

It has recently been reported that central DNA Flap mutant HI-viruses maintain wild-type infectivity in single cycle infection assays following production in APOBEC3G-negative 293T cells [16]. To test the possibility that the apparent discrepancy between our results and those reported by Hu *et al*. 2010 [16] might reflect differences between virus strains or the production protocol, the molecular clones used in Hu *et al*. were obtained from E. Poeschla (Mayo Clinic). The ability of these viruses to infect P4-CCR5 indicator cells was tested in parallel with our wild-type virus and DNA Flap mutants. The mutants from both studies displayed a comparable infectivity defect (up to 10-fold) at all multiplicities of infection tested (Figure 2E).

To test the possibility that the discrepancies between our results and those from Hu *et al*. might result from the use of different indicator cell lines, the GHOST3 cells used in the Hu *et al*. study were obtained also from E. Poeschla and infected in parallel with molecular clones from Hu *et al*. and our wild-type and mutant viruses (Figure 2F and Additional File 2 Figure S2). GHOST3 cells are HOS CD4+ CXCR4+ CCR5+ carrying the green fluorescent protein (GFP) gene under the control of the HIV-2 LTR promoter. This reporter cell line has the advantage that it does not require cell lysis or addition of substrate, and allows for percentages of infected cells to be measured. We note however that percentages of infected cells have a reduced dynamic range (0-100%, Figure 2F), compared with RLU values from P4-CCR5 cells (4-5 log_10_, Figure 2A, C), and do not distinguish single from multiple integration events. The percentage of infected cells was consistently less for DNA Flap mutants compared with wild-type virus (up to 6-fold), but the amplitude of the infectivity defect was reduced or lost at high multiplicities of infection (Figure 2F), where multiple integration events occur. The narrow linear dynamic range of GHOST3 cells may account for the diverging results reported by Hu *et al*. [16].

### Impact of DNA Flap mutations on viral replication

**Figure 3:**
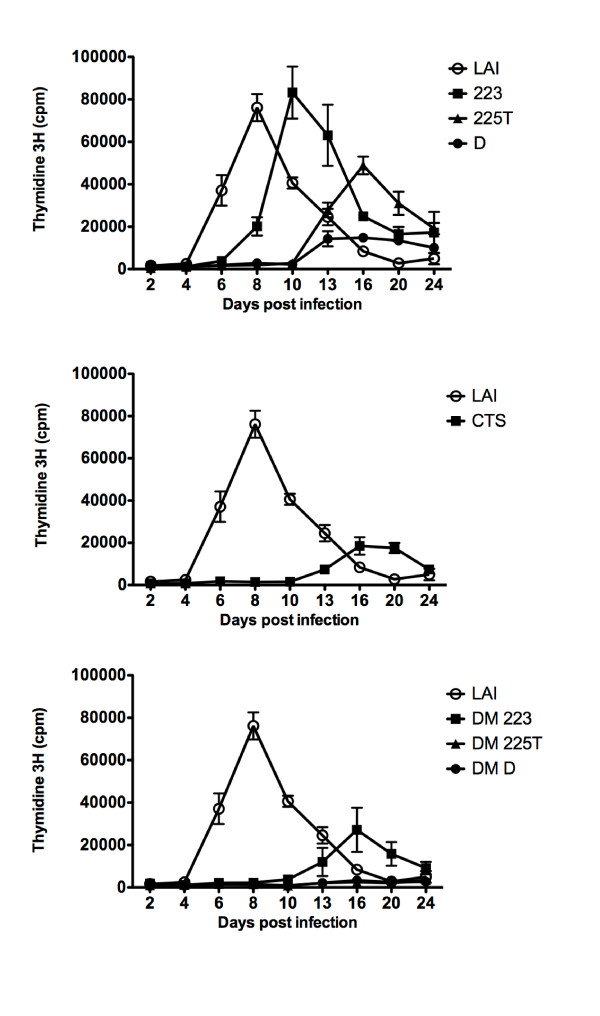
**Replication kinetics in MT4 cells indicate an increased replication defect of DNA Flap double mutant viruses compared with cPPT or CTS single mutants**. MT4 cells were infected with 1 ng p24 of wild-type envelope HIV-1 and virus production was measured at given time points post-infection by performing RT assay on the culture supernatants. Results show the mean of triplicates +/- SEM and are representative of two independent experiments.

Single cycle infection assays are sensitive to very low infectivity and successful integration events as measured by beta-gal activity, but do not guarantee that the viruses can establish a spreading infection in cell culture. We therefore carried out kinetics of replication following infection with wild-type envelope HIV-1. MT4 cells were chosen because they are highly permissible to HIV-1, allowing rapid and efficient replication. MT4 cells are APOBEC3G-negative, and therefore the role of central (+) strand initiation in protecting the genome from editing [14,16] is not a confounding factor in this assay. In 2 independent experiments, the replication of wild-type HIV-1_LAI _peaked at 5-6 days post infection; in contrast, the replication of 223, 225T, CTS and cPPT-D single mutants was delayed on average by 3, 5, 6 and 9 days, respectively, compared with wild-type HIV-1_LAI _(Figure 3). While DM-223 showed a delay similar to that of the CTS mutant, virus replication remained undetectable for DM-225T and DM-D mutants over the 24-day period, indicating that despite minimal single-cycle infection, these viruses do not replicate in the time frame of the assay.

**Figure 4:**
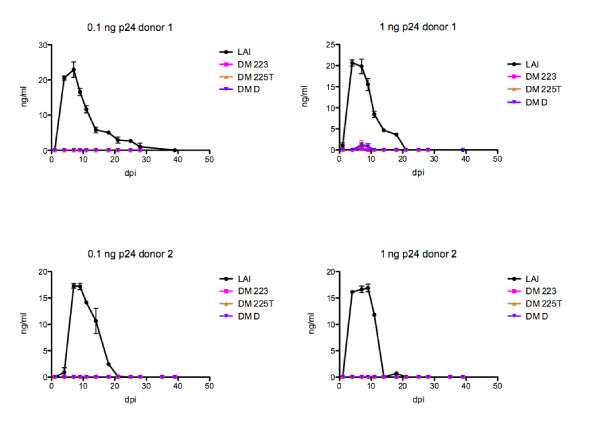
**Replication kinetics in primary PBMCs indicate an inability of double mutant viruses to establish spreading infection**. PBMCs were infected with 0.1 and 1 ng p24 of wild-type envelope HIV-1, and virus production was measured at given time points post-infection by p24 ELISA on the culture supernatants. Results show the mean of duplicates +/- SEM for two donors.

To verify that the observed replication defect is not cell-dependent, we also tested the ability of double-mutant viruses to establish spreading infection in human primary PBMCs from two healthy donors, which were infected with 0.1 and 1 ng p24 of wild-type and double mutant viruses. The replication of wild-type HIV_LAI _peaked at 7-10 days post-infection (Figure 4). In contrast, no peak of replication was detected for double mutant viruses over the 40-day period, and virus concentration was only sporadically measurable above the assay minimum detectable limit (*ca*. 3 ng/ml). The fact that DM 223, which replicated in MT4 cells despite some delay compared to wild-type virus (Figure 3), did not establish spreading infection in PBMCs confirms the high permissiveness of MT4 cells. Moreover, although viruses used for this study were wild-type for Vif, the stronger block in replication observed in PBMCs may also reflect the increased sensitivity of DNA Flap mutants to APOBEC3G/F-mediated deamination in PBMCs [14,16]. We conclude that DNA Flap double mutant viruses cannot establish spreading infection in lymphocytic cell cultures despite detectable levels of infectivity in single cycle infection assays.

### Impact of DNA Flap mutations on nuclear import

**Figure 5:**
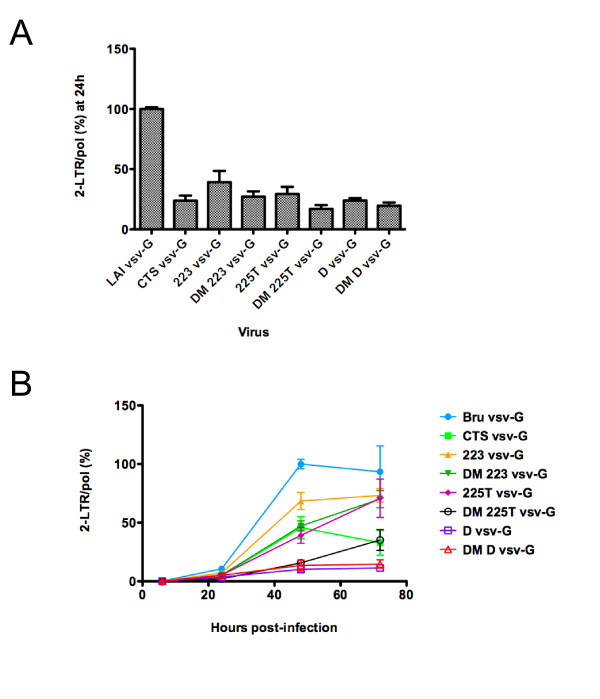
**Quantification of 2-LTR circles and total HIV-1 DNA (Pol) by quantitative PCR shows defects in nuclear import for all DNA Flap mutants**. P4-CCR5 cells were infected with wild-type and DNA Flap mutant viruses pseudotyped with VSV-G envelope. **(A) **Results show the number of 2-LTR circles normalised for *pol *copy numbers at 24 h post-infection. Non-infected samples did not give rise to amplified products. Cells infected in the presence of Nevirapine served as controls for each virus infection (< 10^3 ^copies for all samples) and were subtracted from each value. Values are represented as a percentage of wild-type and are the mean of 3 independent experiments carried out in duplicate +/- SEM. **(B) **Results show 2-LTR circles at given time points post-infection, normalised for *pol *copy number at 24 h post-infection. Values are represented as a percentage of wild-type at 48 h post-infection and are the mean of 2 independent experiments carried out in duplicate +/- SEM.

Once reverse transcribed in the cytoplasm, the HIV-1 linear DNA is imported into the nucleus where it either integrates or circularises. Circular forms of non-integrated viral DNA contain one or two long terminal repeats (LTR) and are thought to be dead-end products of infection. Since they are found exclusively in the nucleus of infected cells, they constitute convenient markers of nuclear import. We therefore used quantitative PCR to measure two-LTR DNA circles [41] in P4-CCR5 cells infected with wild-type, single and double mutant HIV_LAI _vsvG viruses. At 24 h post-infection, quantification of 2-LTR junctions revealed a reduction in 2-LTR copy numbers of 3-6-fold for all single and double DNA Flap mutants compared to wild-type virus, indicating a defect in nuclear import (Figure 5A). As previously observed (P. Charneau, unpublished data), the CTS mutant has a nuclear import defect comparable to cPPT mutants. Mutations in the cPPT lead to linear DNA lacking the central DNA Flap while mutations in the CTS lead to linear DNA containing an aberrantly sized DNA Flap. Since both DNA Flap mutant types have impaired nuclear import, it is the integrity of the DNA Flap structure, rather the presence or not of a (+) strand discontinuity, that accounts for nuclear import. Furthermore, DNA Flap mutant viruses maintained a defect in 2-LTR copy numbers over a 72 h time course (Figure 5B), indicating an overall defect in nuclear import rather than a delay. D vsv-G and DM-D vsv-G viruses remained strongly defective for nuclear import throughout the time course (Figure 5B). Similar to results obtained from infectivity experiments (Figure 2), the combination of cPPT and CTS mutants led to a further decrease in 2-LTR copy numbers compared with single mutants. Using this test, DM-225T and DM-D maintain residual levels of 2-LTR circles of 10-20% of wild-type virus, indicating that disruption of the central DNA Flap severely impairs but does not abrogate nuclear import.

**Figure 6:**
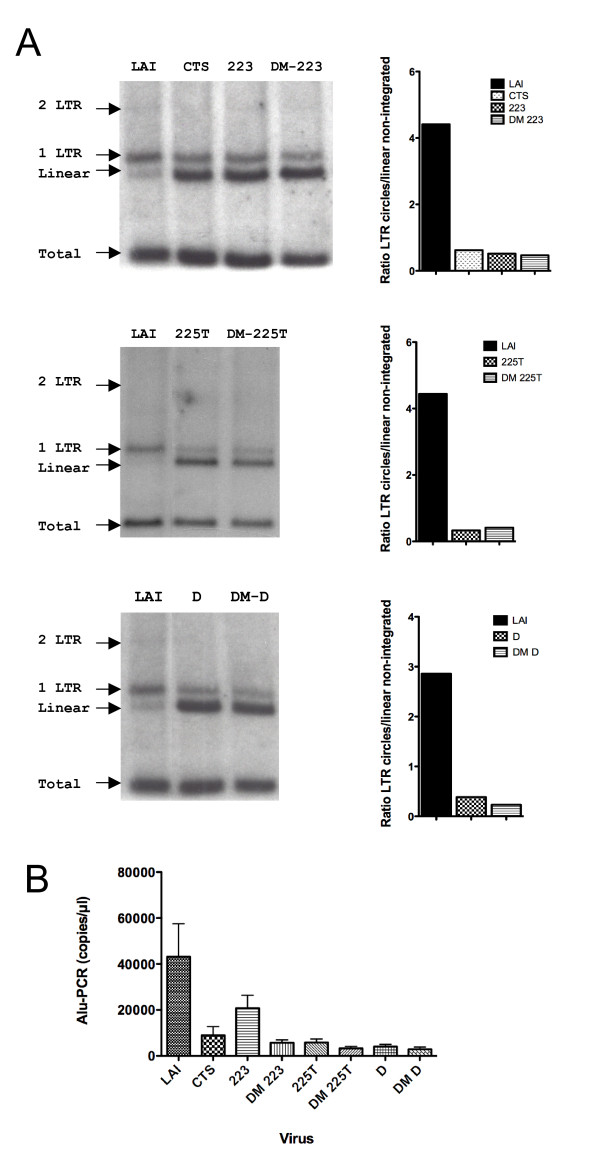
**Quantification of LTR circles, linear unintegrated, and integrated HIV-1 DNA by Southern blotting and Alu-PCR confirms defects in nuclear import for all DNA Flap mutants**. **(A) **MT4 cells were infected with wild-type or DNA Flap mutant viruses pseudotyped with VSV-G envelope. Total DNA was isolated 48 h post-infection and intracellular viral DNA forms were analysed by Southern blotting. Bands were quantified using Phosphorimager and nuclear import was assessed by plotting the ratio of LTR circles (nuclear) to linear non-integrated HIV-1 (predominantly cytoplasmic). **(B) **Assessment of HIV-1 integration by Alu-PCR. The graph shows mean values of 3 independent experiments +/- SEM.

Two-LTR circles represent only a minute fraction of HIV-1 DNA in infected cells (< 1% of nuclear viral DNA), and small variations in their levels will lead to important changes in inferred nuclear import levels. One-LTR circles are more abundant (*ca*. 35% of nuclear viral DNA) [9], but cannot be distinguished by quantitative PCR. We therefore used restriction enzyme analysis coupled to Southern blotting to detect total, linear non-integrated, and circularised forms of HIV-1 DNA in MT4 cells [9]. In three independent experiments, we found that at 48 h post-infection with wild-type HIV-1_LAI_, under 1% of viral DNA was in the form of 2-LTR circles, 35-45% as 1-LTR circles, and 10-15% as linear unintegrated DNA (Figure 6A), which is concordant with previous work [9]. Linear viral DNA molecules undergo rapid integration or circularisation soon after entry in the nucleus [42-45]; therefore, measured linear DNA corresponds predominantly to cytoplasmic viral DNA, and its accumulation is indicative of a nuclear import defect. The ratio of 1- and 2-LTR forms over linear cytoplasmic viral DNA at 48 h post-infection, which was of 4 on average for wild-type HIV-1, was reduced, in the case of mutations in the cPPT, to 0.2-0.6 indicating a nuclear import defect (Figure 6A). Using this Southern blot assay, we did not notice an additional impairment of nuclear import for DM viruses. Furthermore, despite the reduction in nuclear import, nuclear forms of viral DNA (1-LTR) could still be detected for both single and double DNA Flap mutants, confirming the residual level of nuclear import obtained with 2-LTR quantitative PCR.

While these findings suggested that a residual proportion of viral pre-integration complexes might enter the nucleus in the absence of the central DNA Flap, we could not exclude that a small proportion of DNA circles form independently of nuclear import, through auto-integration events in the cytoplasm for instance [45]. To verify this, we quantified integrated viral DNA using Alu-PCR. If viral DNA circularisation occurs independently of HIV-1 nuclear import or at the expense of HIV-1 integration, which is essential for productive infection, integrated viral DNA would not be detected following infection with DNA Flap mutant viruses. We found that cPPT and CTS mutations led to significant reduction in detected integrated HIV-1 genome and that the combination of cPPT and CTS mutations in double mutants led to even greater defects in integration (Figure 6B). The integration defect for the 223 mutant was only 2-fold, which is concordant with the single cycle infectivity and quantitative PCR of 2-LTR circles data (Figure 2). Importantly, integrated signal could be detected for all viruses, including the most disrupted DM-D virus, equivalent to 5-10% of wild-type levels, consistent with the result that all mutant viruses have some residual nuclear import.

Taken together, our data indicate that all DNA Flap mutants analysed have a strongly impaired nuclear import, but maintain a residual level of nuclear import which amounts to 5-15% of wild-type HIV-1, depending on the assay used. The mechanism for this DNA-Flap-independent nuclear import is unknown. The proportion of circularised and integrated HIV-1 following nuclear import is similar to that observed for wild-type virus. However, levels of integrated virus are insufficient to establish spreading infection.

## Discussion

The central DNA Flap is an important determinant of HIV-1 nuclear import, but previously described cPPT mutants still maintain minimal nuclear import (of *ca*. 10% of wild-type virus) resulting in residual infection in single-cycle titrations. The previous lack of sufficiently disruptive cPPT mutants has prevented us from concluding whether these residual levels are due to incomplete disruption of the DNA Flap or whether they point to a DNA-Flap-independent nuclear import. In this study, we combined cPPT and CTS mutations to ensure that possible (+) strand central initiation in 10% of cPPT mutant viruses will not lead to the formation of functional DNA Flap. We found that the most affected double mutants were unable to establish spreading infection in the highly permissive MT4 cells, indicating that the DNA Flap is required for virus replication. Surprisingly, all DNA Flap mutants, including the most disrupted, maintained residual nuclear import levels amounting to 5-15% of wild-type levels.

Our results reveal an apparent discrepancy between the detected residual nuclear import and the absence of spreading infection in lymphocytic cells. A residual proportion of viral pre-integration complexes might enter the nucleus in the absence of the central DNA Flap, but poor efficiency, low numbers or sub-optimal integration may be detrimental for viral replication. It has been suggested that APOBEC editing wholly accounts for the infectivity defect of cPPT mutant HIV [16]. While we cannot exclude a minor contribution of APOBEC editing to the phenotypes we observe, it cannot entirely contribute to the replication defect of DNA Flap mutant viruses since the most defective DNA Flap mutants were unable to establish spreading infection in MT4 cells, which are APOBEC3G-negative.

### The DNA Flap controversy

The central DNA Flap and its importance for HIV-1 replication have been controversial for many years. Its role as a cis-acting determinant of nuclear import has proven all-important for gene transfer based on lentiviral vectors and in the context of replicative viruses [5,9-13,17-21,23,24,26-31,33,34,39]. This defect is independent of envelope tropism (R5, X4, VSV-G) or cell type used (cell lines, primary lymphocytes, APOBEC3G/F status). Still, a certain element of contention remains regarding the extent of the DNA Flap's importance, with some reports contesting its role in nuclear import and replication [16,35-38].

**Figure 7:**
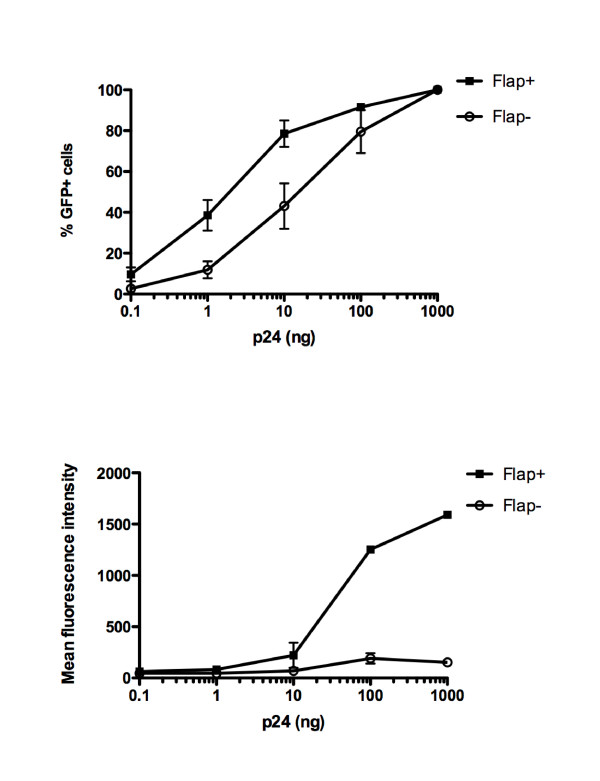
**Effect of the central DNA Flap on percentage of transduced cells and mean fluorescent intensity upon increasing MOI**. HeLa cells were transduced with Flap+ or Flap- HIV-derived vectors (TRIP-eGFP and HR-eGFP, respectively) [9] in 6-well plates with 0.1, 1, 10, 100 or 1000 ng/0.3 × 10^6 ^cells. The percentage of GFP-positive cells and the mean fluorescent intensity were assessed 48 h post-transduction by flow cytometry (BD Calibur).

We believe this controversy may be resolved by considering two points. Firstly, the benefit to infection/transduction brought by the central DNA Flap will be overseen in saturating experimental conditions with assays that cannot distinguish single from multiple infectious events per cell. Concordantly, high MOIs in the context of lentiviral transductions attenuate defects measured in the absence of the central DNA Flap [11]. Similarly, this paper shows that the infectivity defect of DNA Flap mutants is more apparent for wild-type envelope HIV-1 than for VSV-G pseudotyped HIV-1, which is 10-fold more infectious; and more apparent in PBMCs than MT4 cells, which are highly permissive. In transduction experiments using HIV-1 derived vectors coding for eGFP, the benefit of the DNA Flap in terms of the percentage of transduced cells will also decrease with increasing MOI, with transduction efficiencies that can reach 100% for both Flap+ and Flap- vectors in permissive cells (Figure 7). The same loss of a differential phenotype at high and low MOI was also observed following infection of GHOST cells with the viruses from the Hu et al., 2010 study (Figure 2F). In contrast, the difference in mean fluorescent intensity (MFI), which reflects cumulative nuclear entry events, will increase (Figure 7). Therefore, in the case of multiple infectious events per cell, the true impact of the central DNA Flap may only be reliably assessed by combining both percentage of infected cells and MFI [31].

Secondly, DNA Flap mutant viruses display an equal nuclear import defect in both dividing and non-dividing cells [9,10,39,46]. Therefore, assays that compare cycling cells with aphidicolin-arrested cells will invariably see no difference in terms of nuclear import and infectivity of DNA Flap mutant viruses. These experiments are based on the hypothesis that HIV-1 passes through the nuclear pore in non-dividing cells, but uses an alternative route for nuclear entry during mitosis. However, several arguments suggest that this might not be the case. (1) A mitosis-independent nuclear import in cycling cells has been reported [47]. (2) A genome-wide RNA interference-based screen comparing HIV-1 and MLV infections identified unique nuclear import factors for HIV-1 even though the study was carried out in cycling cells [48]. (3) The assumption that HIV-1 might passively gain access to the chromatin upon mitosis, if based on the belief that cytoplasmic and nuclear contents mix homogeneously throughout mitosis, is not valid. Indeed, evidence suggests that mitotic cells maintain spatial information through gradients, such as the RanGTP gradient that surrounds chromatin [49,50]. (4) HIV-1 mutants with a nuclear import defect in cell cycle-arrested cells often maintain this defect in cycling cells [9,10,39,46,51]. (5) The replication of certain lentiviruses (such as EIAV, CAEV and VISNA) is entirely limited to macrophages, which do not divide.

### Mechanism of DNA-Flap-dependent nuclear import

We have previously shown that the central DNA Flap mediates nuclear import by promoting viral capsid uncoating at the nuclear pore [17]. However, the molecular mechanisms underlying DNA-Flap-dependent maturation of HIV-1 capsids remain unknown. Since DNA-Flap-dependent uncoating can be reconstituted *in vitro *in purified vector particles undergoing endogenous reverse transcription, it is not likely that any cellular component(s) is implicated in the uncoating process unless it is already present in the particle. One possible hypothesis is that formation of the central DNA Flap at the end of reverse transcription triggers major morphological rearrangements that lead to the release of the HIV-1 pre-integration complex from the capsid core. Intriguingly, CTS mutations, which lead to DNA Flap structures with aberrant length spread over a distance of 500 nt downstream of the cPPT [6], severely impair nuclear import and infectivity. These data suggest that a central DNA Flap of aberrant length is as detrimental to viral replication as is the absence of the central DNA Flap.

### Mechanism of DNA-Flap-independent nuclear import

The mechanism for the DNA-Flap-independent nuclear import uncovered by this study is unknown, but evidence suggests that it might involve at least some of the same host factors as DNA-Flap dependent nuclear import since Flap-negative viruses are as sensitive to the depletion of TNPO3 or Nup153 as wild-type virus [52,53]. DNA Flap-independent nuclear import cannot be due to viral complexes entering the nucleus via an alternative route during mitosis, since the DNA-Flap-independent nuclear import reported here may be observed in both cycling and non-cycling cells [9,10,39,46]. We have noted that smaller lentiviral genomes, such as minimal HIV-1 derived vectors, have a higher proportion of DNA-Flap-independent nuclear import (P. Charneau, unpublished data), suggesting that the smaller the viral genome, the less dependent it is on the central DNA Flap for passage through the nuclear pore.

Taken together, our data establish that the integrity of the central DNA Flap is required to support a spreading infection and confirm that all cPPT and CTS mutants have a nuclear import defect. Although this defect may vary in its amplitude depending on the nuclear import assay and the MOI used, it is consistently observed. Of note, central (+) strand initiation may also carry further benefits for HIV-1 replication besides assisting viral nuclear import, such as protection from APOBEC3G/F editing as previously shown [14,16]. Here, we show that even the most disrupted DNA Flap mutants still maintain residual nuclear import, but that this does not support spreading infection in human lymphocytic cells. It will be interesting to determine the mechanisms for this DNA Flap-independent nuclear import.

## Methods

### Cells and viruses

The P4-CCR5 reporter cells are HeLa CD4+ CXCR4+ CCR5+ carrying the Lac Z gene under the control of the HIV-1 LTR promoter [6]. MT4 cells are HTLV-1 transformed human CD4+ T cells that allow acute cytopathic HIV-1 infection [54]. The 293T cells are human embryonic kidney cells. Citrate human blood was obtained from healthy donors (Etablissement Français du Sang) and PBMCs were isolated following Ficoll gradient. GHOST3 cells are HOS CD4+ CXCR4+ CCR5+ carrying the green fluorescent protein (GFP) gene under the control of the HIV-2 LTR promoter.

The viral molecular clone used in our study was HIV-1_BRU _also called LAV (Lymphadenopathy-associated virus), based on the 1983 isolate from a homosexual patient with lymphadenopathy [55]. This molecular clone is HIV-1_LAI _for all intents and purposes and is referred to as such in the manuscript. In the case of pseudotyping with VSV-G, we used a Δenv molecular clone that was generated by deletion of the 1.3 kb *Kpn*I-*Bgl*II fragment in HIV-1 *env *[56]. Molecular clones from the Hu et al., 2010 study are NL4-3 Vpr-, WT, mcPPT (cPPT mutant) or DM (cPPT and CTS double mutant).

The generation of 223 [7], 225T [17], cPPT-D [9] and CTS [6] mutants by site directed mutagenesis has been described previously. Double mutants were generated by splice overlap extension PCR using the following primers to amplify the cPPT region, DM1 5'-ACATACAGACAATGGCAG-3' and DM2 5'-TGCTATTATGTCTACTATTC-3' and the following primers to amplify the CTS region, DM3 5'-ATAGTAGACATAATAGCAAC-3' and DM4 5'-TATGTCGACACCCAATTC-3'.

### Virus production

Viruses were produced by transient transfection of 293T cells using calcium phosphate co-precipitation with the wild-type or mutant proviral plasmids based on pLAI or pNL43 Vpr-. In the case of pseudotyping with VSV-G, pLAIΔenv was co-transfected with the VSV-G envelope expression plasmid pHCMV-G [57]. Virus concentration in supernatants was measured by p24 ELISA according to the manufacturer's instructions (Perkin Elmer).

### Quantitative PCR

P4-CCR5 cells were infected with LAI-vsvG viruses (500 ng p24 antigen per 2 × 10^6 ^cells in 2 ml) for 2 h at 37°C and thereafter maintained at a concentration of 0.33 × 10^6 ^cells/ml. For each viral strain, a control of infected cells cultured in the presence of 5 μM nevirapine, a nonnucleosidic RT inhibitor, was included. Total cellular DNA was extracted at given time points post-infection according to manufacturer's instruction (Qiagen) 2-LTR junctions were quantified by real-time PCR using a Realplex instrument (Eppendorf) with 5'AACTAGGGAACCCACTGCTTAAG3' forward primer, 5'TCCACAG ATCAAGGATATCTTGTC3' reverse primer and 5'FAM ACACTACTTGAAGCACTCAAGGC AAGCTTT TAMRA3' probe [58]. *Pol *gene copy number was determined with 5'TTTAGATGGA ATAGATAAGGCCCAA3' forward primer, 5'CAGCTGGCTAACTATTTCTTTTGCTA3' reverse primer and 5'FAM AATCACTAGCCATTGCTCTCCAATTAC TAMRA3'. Amplification was carried out for each reaction in 20 μl with 300 nM of each primer, 200 nM of probe, 5 μl of total-cell DNA and 10 μl of 2X of FastStart Universal Probe Master (Roche). Assessment of integration by Alu-PCR was performed as previously described [59] at 24 h post-infection.

### Restriction enzyme analysis of intracellular viral DNA forms by Southern blotting

MT4 cells were infected with HIV-1_LAI _vsvG viruses (500 ng p24 antigen per 10^7 ^cells in 1 ml) for 2 h at 37°C and thereafter maintained at a concentration of 0.5 × 10^6 ^cells/ml. At 48 h post-infection, total DNA was isolated by lysis in 10 mM Tris, 10 mM EDTA and 0.6% SDS. Samples were treated by RNase A (100 μg/ml) for 1 h at 37°C, and proteinase K (100 μg/ml) for 2 h at 55°C. DNA was extracted by phenol chloroform and digested with *Dpn*I to remove contaminating bacterial plasmid DNA. Viral DNA was then digested with *Msc*I and *Xho*I, which create well-defined fragments on the basis of which the different intracellular forms of viral DNA can be distinguished [9]. Digested samples were then analysed by Southern blotting, using a probe that overlaps one of the two *Msc*I sites in the HIV-1 genome [9]. Southern blots were quantified by phosphorimager (Molecular Dynamics) and the ImageQuant software.

### Single-cycle titration in P4-CCR5 and GHOST3 cells

P4-CCR5 infections were carried out in triplicate in 96-well plates (10,000 cells per well) with increasing doses of LAI or LAI-vsvG. β-Galactosidase activity was measured at 48 h postinfection by using a chemiluminescent β-galactosidase reporter gene assay according to manufacturer's instructions (Roche). GHOST3 infections were carried out in 6-well plates (400,000 cells per well) with increasing doses of LAI or NL43 Vpr-. The percentage of GFP+ cells was assessed 48 h p.i using flow cytometry (BD Calibur).

### Replication kinetics

MT4 cells were infected with HIV-1_LAI _and cPPT/CTS mutants in 96-well plates (1 ng p24 antigen per 5 × 10^4 ^cells in 200 μl). Every 2 days for 24 days, 100 μl of supernatant were collected and replaced by fresh medium. Viral concentration in supernatants was assessed by RT-assay. PBMCs, stimulated for 3 days with Concanavalin A (5 μg/ml), were infected with HIV_LAI _and cPPT/CTS mutants in 96-well plates (1 ng p24 antigen per 1 × 10^5 ^cells in 200 μl). Every 3-4 days for 40 days, 100 μl of supernatant were collected and replaced with fresh medium containing IL2 (final concentration 10 ng/ml). Viral concentration in supernatants was assessed by p24 ELISA (Perkin Elmer).

## Competing interests

The authors declare that they have no competing interests.

## Authors' contributions

CI generated the mutants and carried out Southern blotting experiments. MR and JF performed quantitative PCR. CI, MR, FDN, JF, RG, PS and NJA performed all other experiments. PC conceived the study. NJA participated in the design and coordination of the study, and wrote the manuscript. All authors read and approved the final manuscript.

## Supplementary Material

Additional file 1 **Figure S1: cPPT and CTS mutations do not affect viral production**. The p24 concentrations (ng/ml) are shown for 3 to 6 independent preparations of each virus, both wild-type and VSV-G pseudotyped envelopes. The graph shows mean values +/- SEM. One-way Anova analysis indicated that there is no statistically significant difference between any of the 16 mean values. Click here for file

Additional file 2 **Figure S2: GHOST cells were infected in parallel with viruses**. GHOST cells were infected in parallel with viruses from the Hu et al., 2010 study (WT, mcPPT, DM) and our wild-type and mutant viruses (LAI, D, DM D). The percentage of GFP-positive cells was assessed at 48 h p.i using flow cytometry. Results are representative of three independent experiments. Click here for file
