# Supplementary figures and images for: Residual HIV-1 DNA Flap-independent nuclear import of cPPT/CTS double mutant viruses does not support spreading infection
Source: Retrovirology. 2011 Nov 10;8:92. doi: 10.1186/1742-4690-8-92 (PMC3227589; doi:10.1186/1742-4690-8-92)

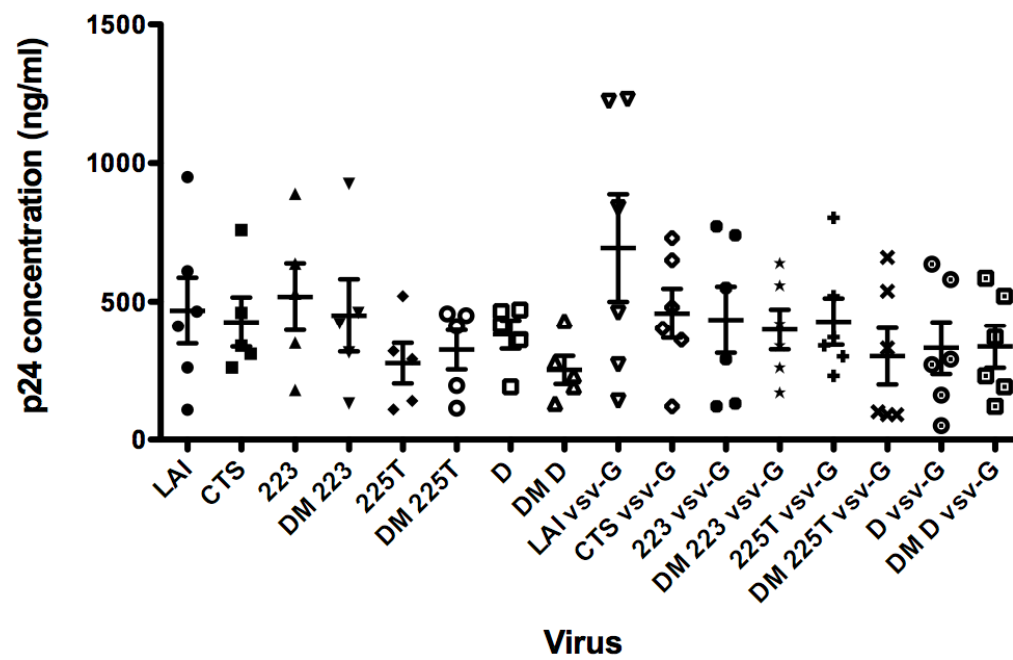

Supplementary Figure 1

Supplement: Additional file 1 — Figure S1: cPPT and CTS mutations do not affect viral production. The p24 concentrations (ng/ml) are shown for 3 to 6 independent preparations of each virus, both wild-type and VSV-G pseudotyped envelopes. The graph shows mean values +/- SEM. One-way Anova analysis indicated that there is no statistically significant difference between any of the 16 mean values. [file 1742-4690-8-92-S1.PDF]

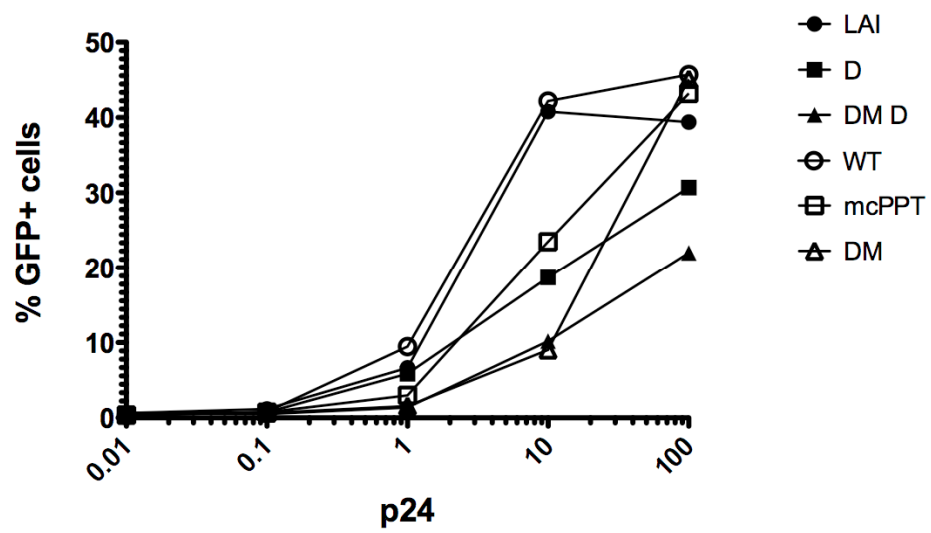

Supplementary Figure 2

Supplement: Additional file 2 — Figure S2: GHOST cells were infected in parallel with viruses. GHOST cells were infected in parallel with viruses from the Hu et al., 2010 study (WT, mcPPT, DM) and our wild-type and mutant viruses (LAI, D, DM D). The percentage of GFP-positive cells was assessed at 48 h p.i using flow cytometry. Results are representative of three independent experiments. [file 1742-4690-8-92-S2.PDF]
